# Expression Pattern of Purinergic Signaling Components in Colorectal Cancer Cells and Differential Cellular Outcomes Induced by Extracellular ATP and Adenosine

**DOI:** 10.3390/ijms222111472

**Published:** 2021-10-25

**Authors:** Clémentine Dillard, Chloé Borde, Ammara Mohammad, Virginie Puchois, Laurent Jourdren, Annette K. Larsen, Michèle Sabbah, Vincent Maréchal, Alexandre E. Escargueil, Elodie Pramil

**Affiliations:** 1 Centre de Recherche Saint-Antoine, Sorbonne Université, INSERM U938, F-75012 Paris, France; clementinedillard@gmail.com (C.D.); chloe.borde_chivot@sorbonne-universite.fr (C.B.); virginie.puchoispisano.pro@gmail.com (V.P.); annette.larsen@sorbonne-universite.fr (A.K.L.); michele.sabbah@inserm.fr (M.S.); vincent.marechal@sorbonne-universite.fr (V.M.); elodie.pramil@hotmail.fr (E.P.); 2 Genomics Core Facility, Institut de Biologie de l’ENS (IBENS), Département de Biologie, École Normale Supérieure, Université PSL, CNRS, INSERM, F-75005 Paris, France; ammara.mohammad@icm-institute.org (A.M.); jourdren@biologie.ens.fr (L.J.); 3 Alliance for Research in Cancerology—APREC, Tenon Hospital, F-75020 Paris, France

**Keywords:** extracellular ATP, 2D/3D cell culture, purinergic receptors, cell death induction, cell cycle arrest, Ca^2+^ mobilization, cyclic nucleotides modulation

## Abstract

The purine nucleotide adenosine triphosphate (ATP) is known for its fundamental role in cellular bioenergetics. However, in the last decades, different works have described emerging functions for ATP, such as that of a danger signaling molecule acting in the extracellular space on both tumor and stromal compartments. Beside its role in immune cell signaling, several studies have shown that high concentrations of extracellular ATP can directly or indirectly act on cancer cells. Accordingly, it has been reported that purinergic receptors are widely expressed in tumor cells. However, their expression pattern is often associated with contradictory cellular outcomes. In this work, we first investigated gene expression profiles through “RNA-Sequencing” (RNA Seq) technology in four colorectal cancer (CRC) cell lines (HT29, LS513, LS174T, HCT116). Our results demonstrate that CRC cells mostly express the A2B, P2X4, P2Y1, P2Y2 and P2Y11 purinergic receptors. Among these, the P2Y1 and P2Y2 coding genes are markedly overexpressed in all CRC cells compared to the HCEC-1CT normal-like colonic cells. We then explored the cellular outcomes induced by extracellular ATP and adenosine. Our results show that in terms of cell death induction extracellular ATP is consistently more active than adenosine against CRC, while neither compound affected normal-like colonic cell survival. Intriguingly, while for the P2Y2 receptor pharmacological inhibition completely abolished the rise in cytoplasmic Ca^2+^ observed after ATP exposure in all CRC cell lines, Ca^2+^ mobilization only impacted the cellular outcome for HT29. In contrast, non-selective phosphodiesterase inhibition completely abolished the effects of extracellular ATP on CRC cells, suggesting that cAMP and/or cGMP levels might determine cellular outcome. Altogether, our study provides novel insights into the characterization of purinergic signaling in CRC.

## 1. Introduction

In addition to its fundamental role in cellular bioenergetics, the purine nucleotide adenosine triphosphate (ATP) plays a crucial role in the extracellular space as a signaling molecule [1,2]. ATP is actively released in the pericellular environment in response to several stimuli, including (1) inflammation-related biological processes, (2) cellular stress and tissue damage during tumorigenesis, (3) cells undergoing apoptosis, and (4) exosomes secreted by cancer cells [3,4,5]. Extracellular ATP can also be secreted during the process of immunogenic cell death induced by chemotherapeutics or released during necrosis [6,7]. This accumulation of extracellular ATP facilitates the recruitment of macrophages and dendritic cells (DCs) by acting as a potent chemotactic stimulus [7,8]. Later, activation of effector T cell responses against cancer-specific antigens occurs, leading to the infiltration of activated T cells and cancer killing cells [9,10]. In healthy tissues, extracellular ATP concentration is very low (in the nM range). However, its concentration can reach hundreds of µM at sites of damaged or inflammatory tissues, as well as in the tumor microenvironment (TME) or at site of metastases [2]. In contrast to the pro-immunogenic role of extracellular ATP, its hydrolysis to adenosine, essentially through the enzymatic activity of the two ectonucleotidases CD39 and CD73, acts as a negative feedback mechanism to prevent excessive immune responses [11,12]. CD39 hydrolyzes both ATP and ADP to AMP, which is further hydrolyzed to adenosine by CD73. The membrane-bound CD73 (or ecto-5′ nucleotidase) is considered as the rate limiting enzyme in the production of extracellular adenosine [13].

Extracellular ATP and adenosine act through their binding to purinergic receptors. Purinergic receptors were first described and characterized in the nervous system, however, it soon became evident that they are also expressed ubiquitously [14,15]. Purinergic receptors can be subdivided into two main subtypes. P1 receptors (P1R) bind to adenosine, while P2 receptors (P2Rs) bind to phosphorylated nucleosides. P1Rs comprise four subtypes (A1R, A2AR, A2BR and A3R) which are all G-protein-coupled, linked to Ca^2+^ mobilization and/or cyclic adenosine monophosphate (cAMP) modulation [2,16]. The P2R family contains seven P2X (P2RX1–7) and P2Y (P2RY1, P2RY2, P2RY4, P2RY6 and P2RY11–P2RY14) members [15,17]. P2X receptors (P2RXs) are ion channels mediating the transmembrane flux of mono- (Na^+^ and K^+^) and divalent (Ca^2+^) cations. P2Y receptors (P2RYs) are coupled via G-proteins to a rise in cytoplasmic Ca^2+^ and cAMP modulation [15]. In the context of cancer, both P1 and P2 receptors are expressed on both tumor and host normal cells [18]. However, several purinergic receptors have been reported to be overexpressed in Human neoplasms, suggesting that some of them might present novel options in terms of therapeutic targets. For example, P2RX7 expression is known to be increased in chronic lymphocytic leukemia [19] as well as in cancers originating from various organs including breast [20], prostate [21], thyroid [22], colon [23,24] and liver [25]. P2RY2 overexpression has been shown in biopsies originating from breast [26] and gastric cancer [27], pancreatic ductal adenocarcinoma [28] as well as of basal cell and squamous cell carcinomas [29]. Finally, A2BR is overexpressed in hepatocellular [30], colorectal [31], oral squamous [32] and bladder urothelial human carcinomas [33].

In addition to their role in immune cell signaling, several reports have shown that high concentrations of extracellular ATP or adenosine can directly act on cancer cells by inducing apoptosis [34,35,36,37]. However, P1R as well as P2R expression in cancer cells has often been associated with contradictory cellular outcomes [2,38,39]. P2RYs have thus been reported to support growth, invasiveness and metastatic spreading suggesting that the increased ATP content in the TME might drive cancer cell proliferation [2,40,41,42]. However, opposite outcomes of P2RY1 or P2RY2 activation have also been described in different settings [43,44]. Similarly, P2RXs play a role in oncogenesis. In particular, P2RX7 has been reported to act on tumor cell growth, cancer cell metabolism, invasiveness, metastatic spreading, angiogenesis and drug resistance [2,45,46]. Accordingly, several small-molecules and biologics directed towards P2RX7 are under development [47]. However, P2RX7 overstimulation by high extracellular ATP concentrations is also capable of inducing tumor cell killing [43]. Thus, as is the case for P2RYs, the P2X7 receptor can either trigger cell death or support tumor cell growth, likely depending on the level of activation of the receptor and the cell type [2].

In this work, we investigated the cellular outcomes induced by extracellular ATP and adenosine in colorectal cancer (CRC) cell lines as well as in non-tumorigenic colonic cells. We focused our work on CRC because of the somewhat contradictory data available so far [47,48]. Moreover, most of the work published to date is mainly focused on two purinergic receptors (e.g., P2RX7 and P2RY2) and only on a few cell lines [23,48,49,50,51,52,53,54]. In order not to limit our study to selected genes, we first explored gene expression profiles through “RNA-Sequencing” (RNA Seq) technology. Experiments were performed on cellular extracts prepared from four different CRC cell lines, including the HT29 cells which are likely the most widely characterized colorectal cancer cells in terms of purinergic signaling [48,49,50,51,52]. The four chosen cell lines show distinct genetic and phenotypic profiles and were grown in either 2D or 3D cell culture to determine whether 3D cell organization might impact the expression profiles of the purinergic signaling components. RNA seq data were then confirmed on selected genes by Real-Time Quantitative Reverse Transcription Polymerase Chain Reaction (qRT-PCR) and compared to the expression levels observed in normal-like colonic cells. Cytotoxic and cytostatic outcomes induced by the exposure to extracellular ATP and adenosine were then assessed in our panel of CRC and normal-like colonic cell lines.

## 2. Results

### 2.1. Transcriptomic Analysis of Colorectal Cancer Cell Lines Grown in 2D and 3D Culture Conditions

**Figure 1:**
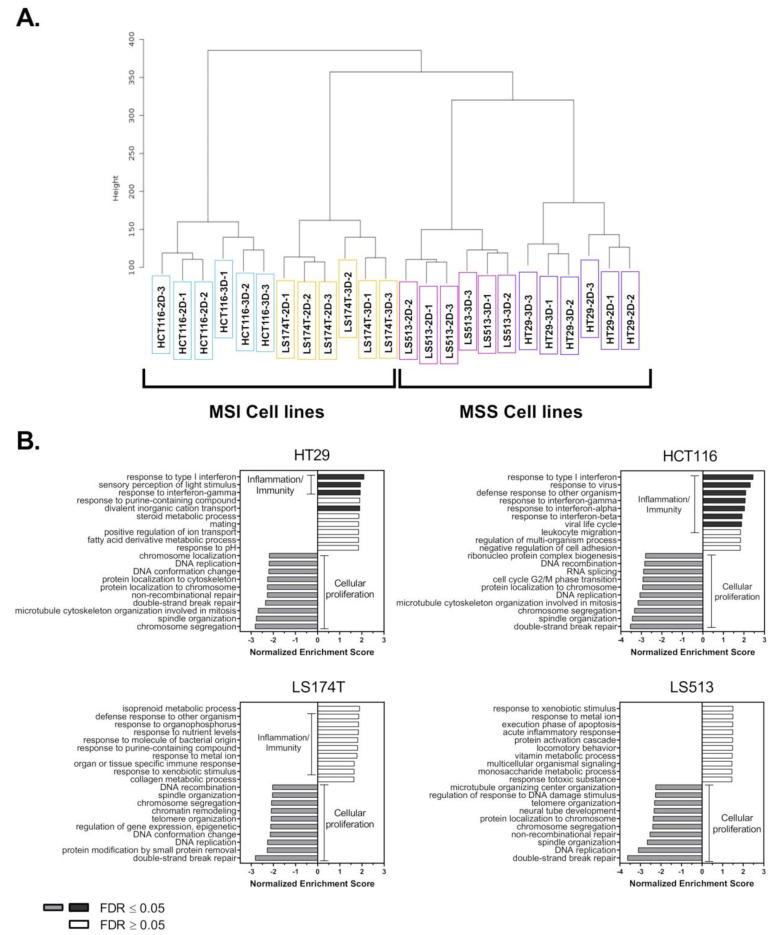
Transcriptomic analysis of colorectal cancer cell lines grown in 2D and 3D culture conditions. (**A**) CRC cell lines (HCT116, HT29, LS513 and LS174T) were cultured in 2D and 3D, RNA extracted and mRNA analyzed through RNA sequencing. Sample data (*n* = 3) were pooled and normalized to compute the cluster dendrogram shown in (**A**). (**B**) Gene set enrichment analysis (GSEA) was performed using the Geneontology, Biological process noRedundant database among the genes significantly overexpressed (Log2foldchange 2Dvs3D > 1) and downregulated (Log2foldchange 2Dvs3D < −0.5) in 3D vs. 2D culture conditions. The significantly enriched and reduced pathways are shown in (**B**).

We first performed gene expression analysis through RNA-Sequencing on samples prepared from our panel of four CRC cell lines grown in either 2D or 3D cell culture conditions. The RNASeq gene expression data (available at www.ncbi.nlm.nih.gov/geo/ (accessed on 12 October 2021) under the accession number: GSE185055) demonstrated that more than 2000 gene expression profiles were affected by the growth conditions for each cell lines (Log2foldchange 2Dvs3D <−0.5 or >1.5 with *p* < 0.05). However, hierarchical clustering showed that even if 2D/3D gene expression profiles diverge for a single cell line, their expression patterns remain similar and samples prepared from either HT29, HCT116, LS174T or LS513 are grouped together (Figure 1A). Our data also demonstrate that hierarchical clustering mostly relies on the Microsatellite stable (MSS)/Microsatellite instable (MSI) phenotypes of the CRC cells, since HT29 and LS174T as well as HCT116 and LS513 gene expression profiles clustered close together.

Gene set enrichment analysis (GSEA) was then performed on the differentially expressed genes between the 2D/3D cell culture conditions (Log2foldchange 2Dvs3D <−0.5 or >1 with *p* < 0.05). As expected, for the four cell lines, GSEA revealed a decreased expression of genes involved in cell proliferation when cells were grown in 3D (Figure 1B). Interestingly, for both HCT116 and HT29, the 3D cell growth condition induced an overexpression of gene sets involved in the interferon and inflammation signaling pathways (Figure 1B). In contrast, no specific gene enrichment profiles reached significance for either LS174T and LS513 cells (false discovery rate (FDR) > 0.05) (Figure 1B).

### 2.2. Transcriptional Expression of Purinergic Receptors and Ectonucleotidases Coding Genes in CRC Cell Lines

**Figure 2:**
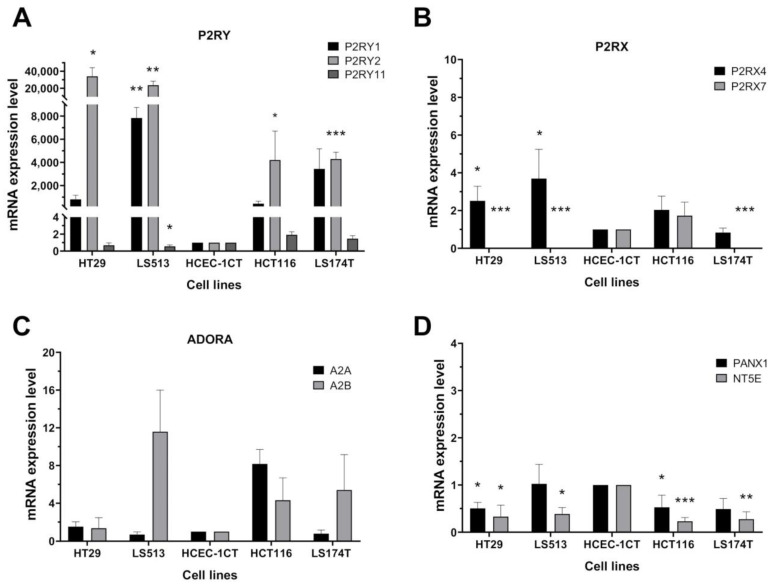
Transcriptional expression of purinergic receptors and ectonucleotidase coding genes in CRC cell lines. The mRNA expression levels for the indicated genes were measured in CRC (HT29, LS513, HCT116 and LS174T) and non-tumorigenic colonic (HCEC-1CT) cell lines (*n* = 4) grown in 2D by quantitative Real-Time PCR and the fold changes calculated by using the 2^−ΔΔCt^ method. Briefly, for each cell line, the mRNA expression levels of the housekeeping gene 36B4 (RPL0) were used an internal control to normalize gene expression. The difference between the Ct values (ΔCt) of the genes of interest and the housekeeping gene was then calculated for each experimental sample, and the difference in the ΔCt values between the experimental (CRC cells) and control (HCEC-1CT) samples ΔΔCt was calculated. Gene expression levels were expressed as the fold changes calculated for each CRC samples compared to the HCEC-1CT cells (2^−ΔΔC^). The mRNA expression of (**A**) ATP metabotropic P2Y receptors, (**B**) ATP ion channel P2X4 and P2X7 receptors, (**C**) adenosine ADORA receptors, and (**D**) the ectonucleotidase CD73 and ATP release hemichannel Pannexin-1 are shown. Data are expressed as the mean +/− SD. *p* values were calculated using Student’s paired *t*-test: * *p* < 0.05, ** *p* < 0.01, *** *p* < 0.005.

To characterize the expression profile of genes involved in extracellular purines signaling and to determine whether 2D/3D cell growth might affect it, we focused on the expression levels of CD39 (ENTPD1), CD73 (NT5E), P1Rs, P2Rs and Pannexin-1 coding genes. Pannexin-1 channel protein is encoded by the PANX1 gene and is involved in the release of ATP from cells. Surprisingly, and independently of the growth conditions, CD39 as well as P2RX1-3, P2RY4-6, P2YR12-14 and A3R coding genes were either not expressed or only slightly expressed in our panel of four CRC cell lines (Tables S1–S4). In contrast, NT5E, PANX1, P2RX4, P2RY1, P2RY2, P2RY11 and A2BR genes were consistently expressed in all the cells tested (Tables S1–S4). Among them, the expression levels of P2RX4 coding gene slightly but constantly increased in the four CRC cell lines when they were grown in 3D (Log2foldchange 2Dvs3D > 0.5 with *p* < 0.05). The effect of the growth conditions was, however, more contrasted for the other genes (Tables S1–S4). Interestingly, the widely studied P2RX7 coding gene was mostly expressed in the HCT116 cells (Table S3) while the P2RX5 coding gene was only expressed in the two MSI CRC cell lines (Tables S3–S4). On the contrary, P2RX6 was mostly expressed in the two MSS CRC cell lines (Tables S1–S2). Finally, only HCT116 cells expressed both A1R and A2AR coding genes (Table S3). To confirm our RNA Seq data, we performed qRT-PCR analysis on extracts prepared from cells grown in 2D. Importantly, for all the genes tested, the cycle threshold (Ct) value calculated showed a good correspondence with the expression levels determined through RNA Seq (Figure S1 and Supplementary Materials). To determine whether the purinergic receptors, Pannexin-1 and ectonucleotidase coding genes might differentially be expressed in CRC cells compared to normal-like colonic cells, we included here mRNA samples prepared from the non-tumorigenic and undifferentiated immortalized epithelial progenitor HCEC-1CT cells [55]. Interestingly, our study revealed that both P2RY1 and P2RY2 coding genes were markedly overexpressed in the four CRC cell lines compared to the HCEC-1CT cells (Figure 2A). In contrast, neither P2RY11 (Figure 2A) nor P2RX4 (Figure 2B) coding gene expression showed marked changes. The A2BR coding gene showed an increased expression level in three out of four CRC cell lines compared to the normal-like cells (Figure 2C), while the CD73 coding gene (NT5E) was slightly but significantly downregulated in CRC cells (Figure 2D). Except for LS513 cells, a similar profile was observed for PANX1 (Figure 2D). Finally, our qRT-PCR analysis confirmed that the P2RX7 coding gene is only expressed at the mRNA level in HCT116 CRC cells (Figure 2B).

### 2.3. Effect of Purine Molecules on 2D Cell Viability and 3D Spheroid Growth

**Figure 3:**
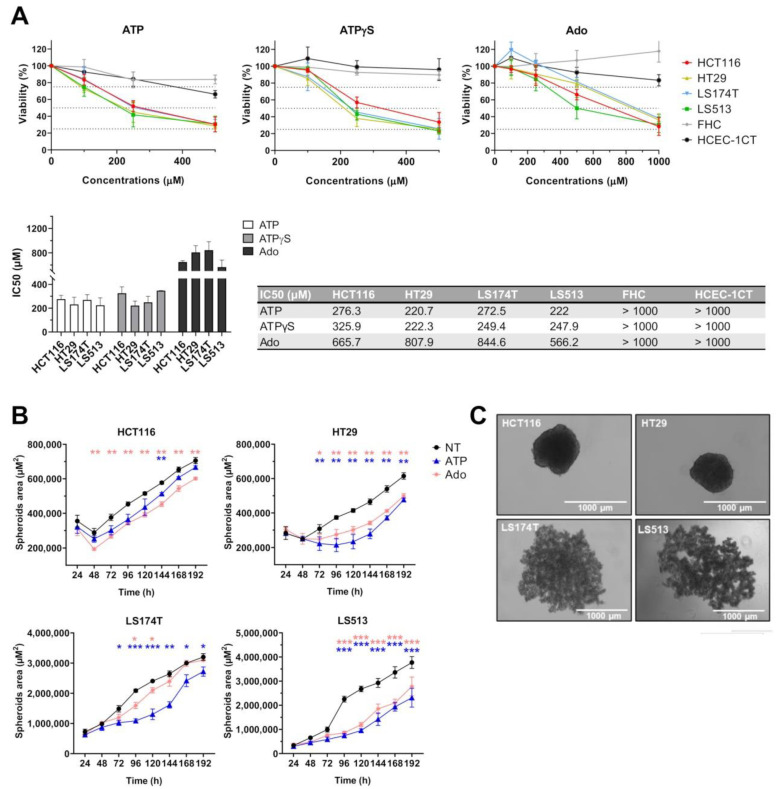
Effect of purine molecules on 2D cell viability and 3D spheroid growth. (**A**) CRC (HCT116, HT29, LS174T, LS513) and non-tumorigenic colonic (FHC, HCEC-1CT) cell lines were treated for four days with increasing concentrations of ATP, ATPγS and adenosine (Ado) (range 0–500 µM) and cell viability assessed by the CellTiter-Glo 2.0 assay (upper panels; *n* ≥ 3). IC50 mean values were calculated and are indicated in the lower panels (histograms and table). (**B**) 3D cell viability assay. CRC (HT29, HCT116, LS174T and LS513) carcinoma cells were cultured in 3D and then treated with 250 µM ATP or 250 µM adenosine (Ado). Growth kinetics was assessed by microscopy (daily pictures) and the area of each spheroid determined by ImageJ. Data are expressed as spheroid area mean +/− SD (*n* = 3); *p*-values were calculated using Student’s paired *t*-test: * *p* < 0.05, ** *p* < 0.01, *** *p* < 0.005. (**C**) Representative images of untreated CRC spheroids taken after 96 h of culture in low attachment plate.

**Figure 4:**
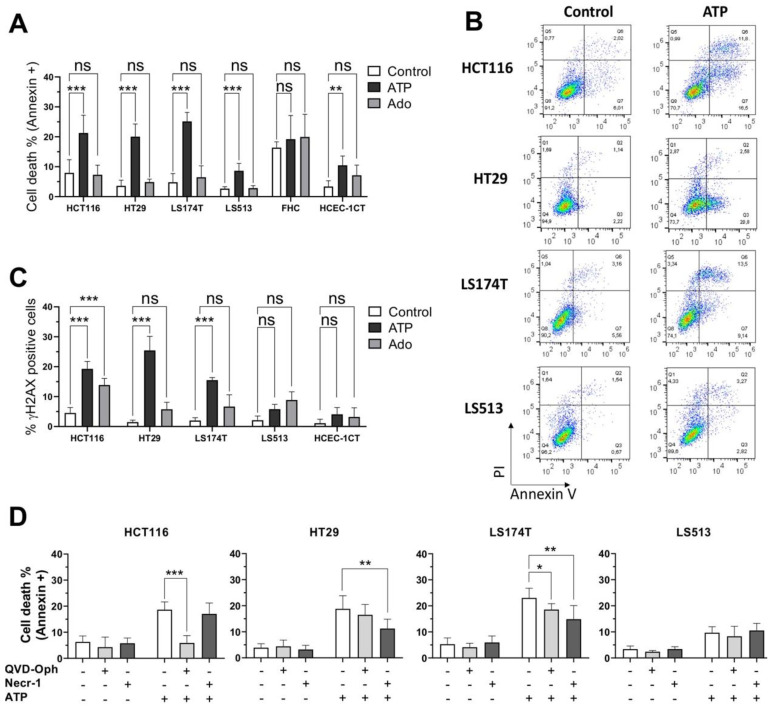
Cell type dependent cellular death processes induced by purine molecules. (**A**) CRC (HCT116, HT29, LS174T, LS513) and normal-like colonic (FHC, HCEC-1CT) cell lines were left untreated (Control) or incubated with 250 μM of either ATP or adenosine (Ado) for 48 h. The percentage of dead cells (Annexin V+) was determined by flow cytometry. Data are expressed as the mean +/− SD (*n* ≥ 3). SDs are indicated by error bars when they exceed symbol size. (**B**) Representative flow cytometry diagrams obtained for CRC cell lines treated or not treated with ATP (250 μM) for 48 h. (**C**) CRC (HCT116, HT29, LS174T, LS513) and normal-like colonic (HCEC-1CT) cell lines were left untreated (Control) or treated with 250 μM of either ATP or adenosine (Ado) for 48 h. The percentage of γ-H2AX positive cells was determined by flow cytometry. Data are expressed as the mean +/− SD (*n* ≥ 3). SDs are indicated by error bars when they exceed symbol size. (*n* = 3). (**D**) CRC (HCT116, HT29, LS174T, LS513) cells were treated (+) or not (−) for 30 min with the pan-caspase inhibitor QVD-Oph (10 µM) or the RIPK1 inhibitor Necrostatin-1 (Necr-1, 40 µM). Cells were then incubated with 250 µM ATP for 48 h. The percentage of dead cells (Annexin V+) was determined by flow cytometry. Data are expressed as the mean +/− SD (*n* ≥ 3). SDs are indicated by error bars when they exceed symbol size. In panels (**A**,**C**,**D**), *p* values were calculated using Student’s paired *t*-test (* *p* < 0.05; ** *p* < 0.01; *** *p* < 0.001).

To begin characterizing the biological effects of extracellular purines on CRC cells, we first assessed the activities of ATP, ATPγS and adenosine on cell viability (Figure 3 and Figure S2). Our results demonstrated that ATP and its non-hydrolysable analog ATPγS show very similar activities on CRC cells (Figure 3A and Figure S2). This observation is consistent with the low level of expression of CD39 on CRC cells as revealed by RNASeq analysis (Tables S1–S4). Importantly, both ATP and ATPγS showed no anti-proliferative activities against two human immortalized colonic cell lines (HCEC-1CT and FHC) (Figure 3A and Figure S2). This suggests that extracellular ATP preferentially targets tumor cells. Similarly, adenosine preferentially affects CRC cells viability (Figure 3A and Figure S2). However, even if higher adenosine concentrations were generally required to reach 50% of growth inhibition when compared to the concentrations used for ATP and ATPγS, the comparison in terms of IC50 value was dependent on the cell viability assay used (compare Figure 3A and Figure S2). This discrepancy is likely due to the fact that both CellTiter Glo Luminescent (Figure 3A) and 3-(4,5-dimethylthiazol-2-yl)-2,5-diphenyltetrazolium bromide (MTT) (Figure S2) assays evaluate the cell viability of proliferating cells by measuring different metabolic alterations induced by the tested compounds [56]. Nevertheless, in all cases, non-tumorigenic cell proliferation was not affected by extracellular purines (Figure 3 and Figure S2).

Because 2D cell cultures have limitations that might impact the response to tested compounds, we evaluated the capability of both extracellular ATP and adenosine to inhibit the growth of 3D tumor cell spheroids, which are believed to more closely mimic in vivo conditions (Figure 3B). Interestingly, the anti-proliferative activities of both purines were confirmed for the HT29, LS513 and LS174T cell lines, with a marked slowdown of spheroid growth. For HCT116 cells, the effect of both purines, while significant, was less marked in comparison with the three other cell lines. This result is likely due to the very tight density of HCT116 spheroids limiting purine diffusion within the 3D structure (Figure 3C). However, these results again confirm that both purines can act on CRC growth in vitro.

### 2.4. Cell Type Dependent Cellular Death Processes Induced by Purine Molecules

Because cell viability assays and spheroid growth kinetics did not allow us to strictly distinguish between a cytotoxic effect and a cytostatic one, we evaluated the capability of both ATP and adenosine to induce cell death in 2D cell culture conditions (Figure 4). We evaluated both compounds at an equimolar concentration corresponding to the IC50 value determined for ATP (250 µM) through the CellTiter Glo Luminescent assay (Figure 3A). To evaluate whether the changes in cell viability were associated with a subsequent induction of cell death, HCT116, HT29, LS174T and LS513 cells as well as the two human immortalized colonic FHC and HCEC-1CT cell lines were treated for 48 h with either ATP or adenosine, and the percentage of Annexin V/propidium iodide (PI) positive cells was determined (Figure 4A,B). Importantly, our results first confirmed the absent (FHC) or minor (HCEC-1CT) cytotoxic effect of both purines on non-tumorigenic cells. However, at 250 µM, only the ATP molecules were capable of inducing cell death in the four CRC cell lines tested, while adenosine did not impact cell survival (Figure 4A). These data confirmed the observation made through the CellTiter Glo Luminescent viability assay, suggesting that ATP is more cytotoxic than adenosine towards cancer cells. Interestingly, while the cell viability of LS513 cells was markedly affected by ATP (Figure 3A), cell death was only moderately induced in these cells, suggesting that ATP treatment is poorly cytotoxic for these cells. To further confirm cell death induction, we evaluated γ-H2AX labeling in cells treated for 48 h with either 250 µM ATP or adenosine (Figure 4C). Beside its canonical function in the DNA damage response processes, Ser-139 phosphorylation of the histone variant H2AX is indeed involved in both apoptosis and caspase-independent programmed necrosis (necroptosis) [57,58,59]. Importantly, γ-H2AX labeling showed very similar patterns to those seen for Annexin V/PI, suggesting that cell death induced by extracellular ATP implies DNA damage in HT29, HCT116 and LS174T cells. Surprisingly, while no Annexin V/PI staining was detected in HCT116 cells after treatment with 250 µM of adenosine, the cells showed clear labeling for γ-H2AX (Figure 4C). This suggests that adenosine treatment might induce DNA damage independent of cell death induction in HCT116 cells.

To further characterize the cell death processes induced by extracellular ATP, CRC cells were treated for 48 h with 250 µM ATP given in the absence or presence of QVD-Oph (a pan-caspase inhibitor with potent anti-apoptotic properties) or Necrostatin-1 (an RIPK1 inhibitor with potent anti-necroptosis activities) (Figure 4D). Interestingly, ATP-induced cell death was markedly inhibited by QVD-Oph in HCT116 cells, suggesting that extracellular ATP acts through apoptosis in these cells. In contrast, the co-incubation of ATP with QVD-Oph had no effect on the cellular outcome of HT29 cells. In this model, only necrostatin-1 partly impaired cell death induction, suggesting that HT29 cells induce necroptosis in response to extracellular ATP. For the LS174T cells, the picture was more complex, since ATP-induced cell death was partially inhibited by QVD-Oph and Necrostatin-1. This suggests that the cellular outcome of the LS174T cells following treatment with extracellular ATP involves induction of both apoptosis and necroptosis. Finally, the two drugs did not impact the cellular outcome of LS513 cells, suggesting that other pathways might be involved in the low level of cell death induction observed here.

### 2.5. Cell Cycle Modulation in CRC Cell Lines Induced by Purine Molecules

**Figure 5:**
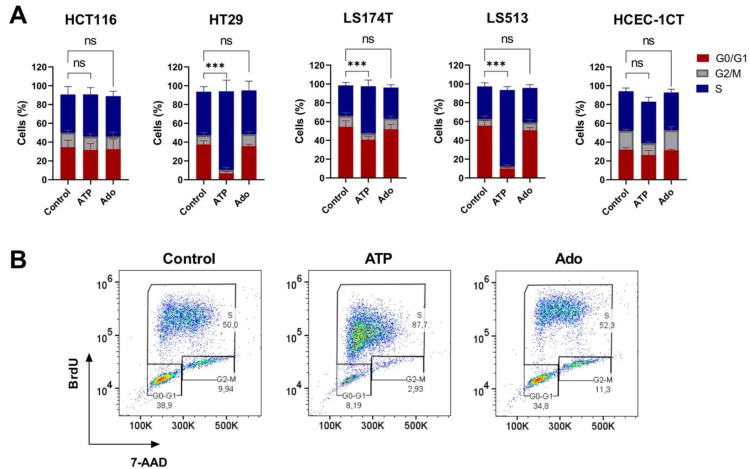
Cell cycle modulation in CRC cell lines induced by purine molecules. (**A**) CRC (HCT116, HT29, LS174T, LS513) and normal-like colonic (HCEC-1CT) cell lines were left untreated (Control) or incubated with 250 μM of either ATP or adenosine (Ado) for 24 h. Prior to flow cytometry analysis and 7-AAD labeling (DNA), living cells were pulse labeled with BrdU. The percentage of cells in the G0/G1, S and G2/M phases was quantified and expressed as a plot. Data are expressed as the mean +/− SD (*n* = 5); *p*-values were calculated using Student’s paired *t*-test (* *p* < 0.05; ** *p* < 0.01; *** *p* < 0.001). (**B**) Representative flow cytometry diagrams for HT29 cells treated or not treated (control) with 250 µM of either ATP or adenosine (Ado). Percentages refer to HT29 cells in G0/G1, S or G2/M.

To determine whether treatment with extracellular purines affects cell cycle progression in CRC cells as well as in the HCEC-1CT cell line, we double labelled cells with BrdU and 7-AAD after 24 h of treatment with 250 µM of extracellular ATP or adenosine (Figure 5). Again, exposure to both adenosine and ATP did not impact the cellular outcome of the non-tumorigenic cells (Figure 5A, right panel). Moreover, in all cases, adenosine treatment showed no effect on the cell cycle progression of CRC cells, confirming the observation made through the CellTiter Glo Luminescent viability assay that higher adenosine concentrations are required to significantly act on tumor cell growth when cells are grown in 2D. However, while exposure to extracellular ATP did not affect the cell cycle progression of HCT116 cells, it significantly increased the percentage of HT29, LS174T and LS513 cells in S-phase, with a marked effect for both HT29 and LS513 (Figure 5A). Interestingly, the flow cytometry dot plots obtained by the double labelling of the cells with BrdU and 7-AAD showed that the increased number of BrdU positive HT29, LS513 and LS174T cells was accompanied by a lower average incorporation of BrdU in individual cells (Figure 5B and Figure S3B,C). This suggests that replication still occurs in individual cells, however, at a slower rate of nucleotide incorporation. Importantly, the effect seen here for the LS513 cell line suggests that the reduced cell viability for these cells observed above (Figure 3A) mostly relies on a cytostatic effect induced by extracellular ATP.

### 2.6. Effects of P2RY1 or P2RY2 Pharmacological Inhibition on Intracellular Ca^2+^ Mobilization Induced by Extracellular ATP

**Figure 6:**
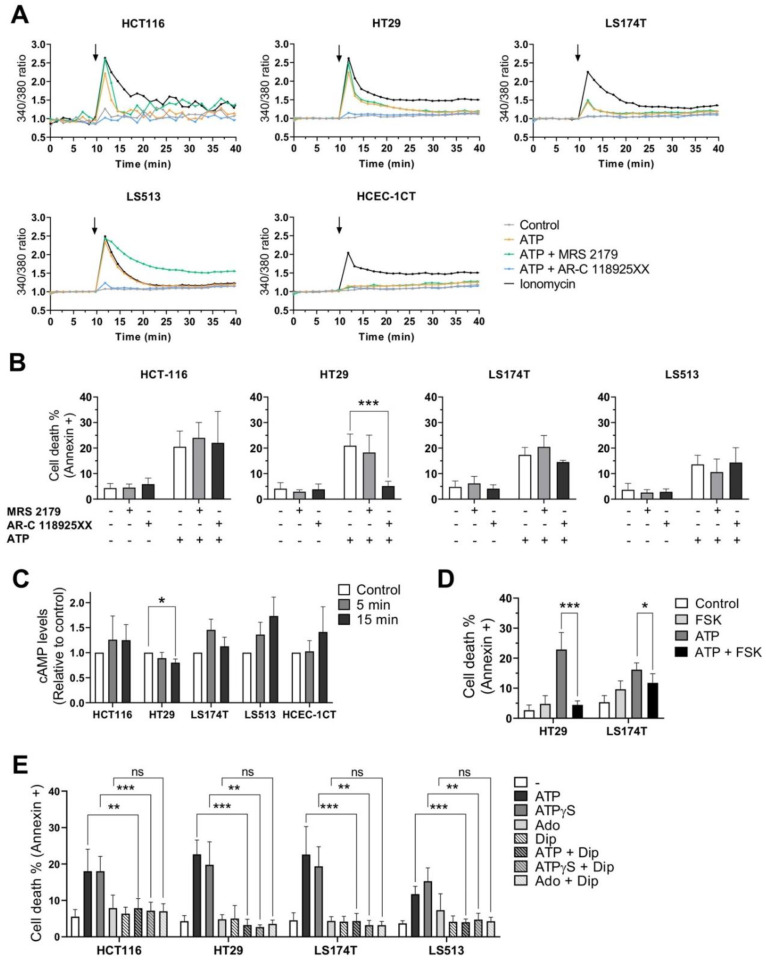
Ca^2+^ mobilization and cAMP levels modulation in CRC cell lines induced by purine molecules. (**A**) Intracellular Ca^2+^ mobilization measured by the Fura-2 AM dye in CRC (HCT116, HT29, LS174T, LS513) and non-tumorigenic colonic (HCEC-1CT) cells. Cells either were or were not pre-incubated with P2RY1 or P2RY2 inhibitors (MRS 2179, 10 µM and AR-C 118925XX, 4 µM, respectively). After 10 min, 250 µM ATP or 1 μM ionomycin (positive control) was added to our samples. Control means untreated cells (negative control). Arrows show the time of ATP/ionomycin injection. Data are expressed as the mean of four independent experiments. (**B**) CRC (HCT116, HT29, LS174T, LS513) cells were treated (+) or not (−) for 30 min with P2RY1 inhibitor (MRS 2179, 10 µM) or P2RY2 inhibitor (AR-C 118925XX, 4 µM). Cells were then incubated with 250 µM ATP for 48 h. The percentage of dead cells (Annexin V+) was determined by flow cytometry. Data are expressed as the mean +/− SD (*n* ≥ 3). (**C**) cAMP levels were measured in CRC and normal-like colonic cells treated or not (Control) for 5 or 15 min with 250 µM ATP. Data are expressed as the mean +/− SD (*n* = 4). (**D**) CRC (HT29, LS174T) cells were treated or not for 30 min with the Adenylyl cyclase activator (FSK, 100 µM) and then incubated with 250 µM ATP for 48 h. The percentage of dead cells (Annexin V+) was determined by flow cytometry. Data are expressed as the mean +/− SD (*n* = 4). (**E**) CRC (HCT116, HT29, LS174T, LS513) cells were treated or not for 30 min with Dipyridamole (Dip, 10 µM) and then incubated with either 250 µM ATP, 250 µM non-hydrolysable ATP (ATPγS) or 250 µM Adenosine (Ado) for 48 h. The percentage of dead cells (Annexin V+) was determined by flow cytometry. Data are expressed as the mean +/− SD (*n* ≥ 4). In panels B, D and E, *p* values were calculated using Student’s paired *t*-test; in panel C, *p*-values were calculated using the Mann–Whitney test (* *p* < 0.05; ** *p* < 0.01; *** *p* < 0.001).

Because of the high expression levels of both P2Y1 and P2Y2 receptors revealed on CRC cells compared to the normal-like HCEC-1CT cell line (Figure 2), we wondered whether these two receptors might be involved in the selective response to extracellular ATP observed for the four CRC cell lines. To assess this, we first measured the rise of cytoplasmic Ca^2+^ induced by extracellular ATP (250 µM) in the presence or absence of two selective competitive antagonists of either P2RY1 (MRS 2179) or P2RY2 (AR-C 118925XX) (Figure 6A). In agreement with the absence of effect of extracellular ATP on the HCEC-1CT cells viability, as well as with the low expression levels of both receptors in these cells, our results showed that ATP exposure here did not induce any sort of Ca^2+^ rise, while our positive control, ionomycin, did. On the contrary, for the four CRC cell lines, ATP treatment led to a fast and significant increase of intracellular Ca^2+^ which was fully abolished by pre-incubating cells with the P2RY2 inhibitor AR-C 118925XX. This, combined with the absence of any effect of MRS 2179, suggests that P2RY2 might be primarily involved in the response of CRC cells towards extracellular ATP.

### 2.7. Effects of P2RY1 or P2RY2 Pharmacological Inhibition on CRC Cell Survival after Exposure to Extracellular ATP

To determine whether the inhibition of intracellular Ca^2+^ mobilization might affect the cellular outcome of CRC cells in terms of cell viability, the four CRC cell lines were treated for 48 h with 250 µM ATP in the presence or absence of MRS 2179 or AR-C 118925XX. The percentage of Annexin V/propidium iodide (PI) positive cells was then determined (Figure 6B). In agreement with the absence of effect of MRS 2179 on Ca^2+^ mobilization, no effect of the drug could be found in terms of cell survival. However, while pharmacological inhibition of the P2Y2 receptor completely abolished the rise of cytoplasmic Ca^2+^ induced by ATP for our four CRC cell lines (Figure 6A), the co-incubation of AR-C 118925XX with extracellular ATP only impacted the cellular outcome of HT29 cells (Figure 6B and Figure S4). These results suggest that, except for HT29 cells, neither P2RY2 nor rapid Ca^2+^ mobilization is likely to play a major role in extracellular ATP signaling leading to cell death induction in CRC cells.

### 2.8. Effects of Extracellular ATP Exposure on Cellular cAMP Levels

In addition to the rise of cytoplasmic Ca^2+^, exposure to extracellular purines is often accompanied by cAMP level modulation [15]. To evaluate whether treating cells with extracellular ATP might affect cAMP levels in CRC as well as HCEC-1CT cells, we measured the immediate change in cAMP intracellular levels occurring after treatment with 250 µM ATP (Figure 6C). As seen previously, only the HT29 cells showed a specific profile in response to extracellular ATP; this was characterized by a significant decrease in cAMP levels. However, for both HCT116 and LS174T the profiles were more contrasted, while cAMP levels seemed to increase for LS513 and HCEC-1CT cells after treatment with ATP. To determine whether the pharmacological modulation of cAMP levels might nevertheless impact the cellular outcome induced by ATP at later time points, the four CRC cell lines were treated for 48 h with 250 µM ATP in the presence or absence of forskolin (FSK), a common activator of adenylyl cyclase (AC, Figure 6D). Again, the HT29 cells showed a clear pattern, demonstrating that FSK can abolish cell death induction triggered by extracellular ATP exposure, likely by increasing intracellular cAMP concentration (Figure S5). This result is in agreement with the fact that ATP exposure led to a decrease in cAMP levels in HT29 cells (Figure 6C), and suggests that modulation of intracellular levels of both Ca^2+^ and cAMP may impact the cellular outcome of this specific cell line. Interestingly, a similar tendency was observed for the LS174T cell line (Figure 6D and Figure S5), suggesting that cAMP level modulation might have a more general impact on outcomes in CRC cells. Unfortunately, because FSK alone was cytotoxic for both HCT116 and LS513 cells (Figure S6), it was not possible to extend this observation to these two cell lines.

### 2.9. Effects of Dipyridamole on CRC Cell Survival after Exposure to Extracellular Purines

To further study the role of 3′,5′-cyclic nucleotide messengers in the response of CRC cells towards extracellular purines, we used the non-selective phosphodiesterase inhibitor dipyridamole (DIP) to modulate intracellular levels of both cAMP and cyclic guanosine monophosphate (cGMP) [60,61]. However, because DIP is also a known inhibitor of adenosine transporters [62] and because it has been reported that DIP can impair the growth inhibitory effect of extracellular ATP by blocking adenosine uptake in human gastric carcinoma HGC-27 cells [63], we used both non-hydrolysable ATP (ATPγS) and adenosine as controls (Figure 6E). As shown before, when the four CRC cell lines were treated for 48 h with 250 µM adenosine, no cell death induction could be detected by Annexin V/PI double labeling. These negative profiles were not impacted by co-treatment with DIP. On the contrary, both extracellular ATP and ATPγS induced a marked increase in dead cells for the four CRC cell lines, which was completely abolished in the presence of DIP. Together, these data confirm that the growth inhibitory effect of extracellular ATP is not mediated by its biotransformation to adenosine or adenosine uptake, and further demonstrate that the effect of extracellular ATP can be circumvented by an increase in cyclic nucleotides (e.g., cAMP/cGMP) following non-selective PDE inhibition in CRC cells.

## 3. Discussion

The direct and indirect anticancer activity of extracellular ATP has long been demonstrated [64]. The initial understanding of these effects on tumor cell survival and immune cell recruitment led to the development of pharmacological strategies aiming at increasing its pericellular concentration or targeting specific receptors [64]. However, the final outcome of such approaches can be difficult to predict, and likely depends on tumor cell types.

First, targeting a single purinergic receptor with selective agonists or inhibitors relies on the expression of well-defined targets. However, purinergic receptor expression varies between cancer cells [15]. In this sense, our data demonstrate that CRC cells mostly express A2B, P2X4, P2Y1, P2Y2 and P2Y11 receptors, while only few transcripts of P2RX7 can be detected in our four CRC cell lines. This observation, as well as those from others [50,65] is of interest since P2RX7 is often referred to as being widely expressed on CRC cells [15] and pharmacological strategies aiming at activating or inhibiting P2RX7 have been proposed [47,66]. Obviously, this discrepancy can be explained by differential controls acting on either gene expression or protein stability [43]. However, even in patient samples, P2RX7 expression is inconsistent and mostly associated with advanced stages of the disease [23,24,47]. This suggests that P2RX7 expression might occur late in the process. Interestingly, our gene expression data show that both P2RX7 and PANX1 coding genes whose products are known to participate to the release of ATP in the extracellular compartment [64] were both downregulated in CRC cells compared to the normal-like HCEC-1CT cell line (Figure 2B,D). This observation, combined with the fact that pro-inflammatory signaling pathways were overexpressed in all our CRC cells when structured in 3D (Figure 1B), suggest that microenvironment-driven cancer cell selection might drive CRC cells, at least initially, to downregulate pathways capable of releasing additional pro-inflammatory molecules, including ATP [67]. This assumption is of particular interest if we consider the differential function of acute and chronic inflammation in tumor progression [68]. Indeed, while chronic inflammation is involved in immunosuppression, the release of extracellular ATP as well as other damage-associated molecular patterns (DAMPs) can trigger acute inflammatory reactions, leading to anti-tumor immune responses at early stages of the disease [7]. Intriguingly, P2RX7 and Pannexin-1 are closely interconnected in this context and were both found to co-immunoprecipitate in HEK293 cells [69,70]. Therefore, their downregulation might allow for maintaining the low extracellular ratio of ATP/adenosine associated with the chronic phase of inflammation [7]. Later in the process of tumorigenesis, P2RX7 overexpression may, on the other hand, help cancer cells to invade, metastasize and resist drugs [2,45,46]. Further studies will be required to determine whether such a dynamic model might apply to CRC.

Another important feature of our study is that among the five purinergic receptors coding genes consistently expressed in CRC, only the P2RY1 and P2RY2 coding genes were markedly overexpressed in all CRC compared to the normal-like colonic cells. The overexpression of the two P2Y1 and P2Y2 receptors might render cancer cells hypersensitive to their extracellular ligands (ADP and ATP or UTP, respectively) as this was reported for other receptors like EGFR [71,72]. Moreover, the tri-phosphorylated nucleotide P2Y2 receptor is known to play an important role in cancer progression, notably for CRC [73,74]. Differential gene expression analysis made through the GEPIA2 web server (http://gepia2.cancer-pku.cn/#index, accessed on 12 October 2021) confirms that P2RY2 is overexpressed in both colon (Log2foldchange TumorvsNormal = 0.996 with *p* = 3.26e^−12^) and rectal (Log2foldchange TumorvsNormal = 1.086 with *p* = 1.97e^−9^) adenocarcinomas when compared to their normal counterparts [75]. Obviously, this makes P2RY2 interesting as a therapeutic target [76]. Accordingly, recent studies have demonstrated that P2RY2 expression was elevated in pancreatic ductal adenocarcinoma and in head and neck squamous cell carcinoma (HNSCC), and its inhibition suppressed cancer cell growth both in vitro and in vivo [28,77]. In our hands, however, the whole picture is less clear and suggests that the observations made in pancreatic cancer and HNSCC might not apply to CRC. Indeed, while our data clearly demonstrated that P2RY2 plays a unique role in Ca^2+^ mobilization when CRC cells were exposed to extracellular ATP (Figure 6A), its selective pharmacological inhibition either had no effect (HCT116, LS174T, LS513) or reversed (HT29) the effects of ATP on CRC cell survival (Figure 6B). Therefore, inhibiting P2RY2 in CRC might be either useless or counter-productive in terms of cancer cell survival. However, because Ca^2+^ mobilization as well as extracellular ATP exposure and P2RY2 activation have been involved in metastasis, cell cycle progression, cell motility and drug resistance [2,28,40,41,42,73,74,76,77,78,79,80,81,82], it would be interesting to further explore the effects of P2RY2 inhibition on these specific cellular processes associated with cancer progression.

Third, while all CRC cells are sensitive to extracellular ATP exposure in terms of growth inhibition, we show here that the cellular outcome clearly depends on the cell line tested. Extracellular ATP can indeed induce apoptosis, necroptosis, a combination of both, or cell cycle arrest (Figure 4 and Figure 5). Importantly, no effect could be identified on the cell viability of two normal-like colonic cells. However, while previous reports suggested that the effect of extracellular ATP might be driven by its biotransformation to adenosine through the action of the two CD39 and CD73 ectonucleotidases [43,83], our results do not support this hypothesis or its generalization to CRC. Indeed, our results show that (i) CD39 is expressed poorly or not at all in our panel of four CRC cell lines (Tables S1–S4); (ii) ATP and its non-hydrolysable analog ATPγS show similar activities in 2D cell viability assays (Figure 3A, Figure 6E and Figure S2); and (iii) adenosine exposure does not induce any form of cellular death when used at the same concentration as ATP (Figure 4A and Figure 6E).

**Figure 7:**
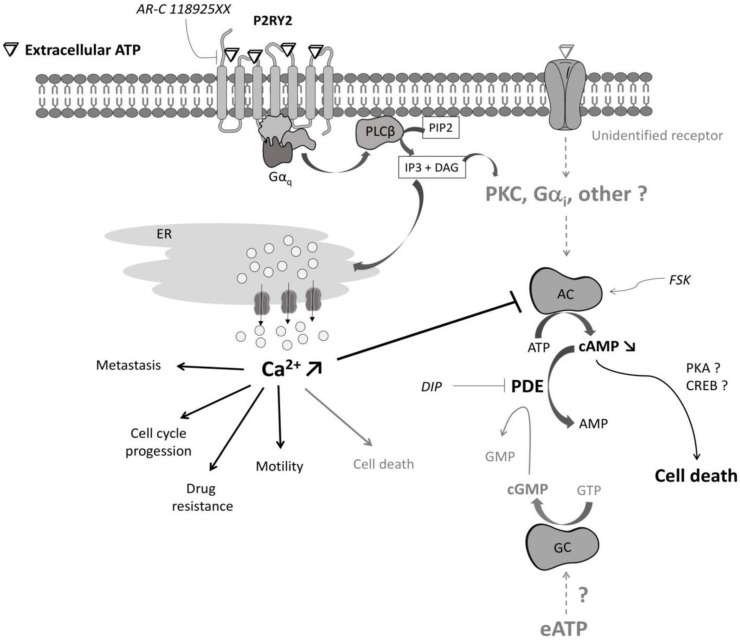
Model depicting how extracellular ATP might affect CRC cell viability. The tri-phosphorylated nucleotides P2Y2 receptor is overexpressed in CRC cells. In the presence of extracellular ATP (eATP), P2RY2 activation induces a rise of cytoplasmic Ca^2+^ which is fully abolished by the selective pharmacological inhibitor AR-C 118925XX. For HT29 cells, extracellular ATP, through its binding to P2RY2, phospholipase C activation and Ca^2+^ mobilization, leads to adenylyl cyclase (AC) inhibition and cAMP level decrease. Subsequently, the decrease in cAMP levels might impact PKA and/or CREB activities and modulate HT29 cell survival. Both the common activator of adenylyl cyclases, forskolin (FSK), and the non-selective phosphodiesterase (PDE) inhibitor, dipyridamole (DIP), can fully abolish the effects of extracellular ATP on HT29 cells. For HCT116, LS513 or LS174T cells, extracellular ATP also induces a rise of cytoplasmic Ca^2+^ through P2RY2 binding. However, Ca^2+^ mobilization does not impact cell viability. This suggests that P2RY2 activation might be involved in the modulation of other Ca^2+^-dependent pathways implicated in cancer progression. Besides its binding to P2RY2, extracellular ATP might act on HCT116, LS513 or LS174T cellular viability through its binding to as-yet unidentified purinergic receptors and the subsequent activation of downstream signaling pathways able to modulate adenylyl and/or guanylyl cyclase (GC) activity. DIP, by increasing both cAMP and cGMP levels, can fully abolish the effects of extracellular ATP on HCT116, LS513 and LS174T cell viability.

Fourth, while the pharmacological inhibition of the P2Y2 receptor completely abolished the rise in cytoplasmic Ca^2+^ observed in all CRC cells after treatment with ATP (Figure 6A), Ca^2+^ mobilization seemed only to impact the cellular outcome of the HT29 cells (Figure 6B). Of note, this observation confirms previous data reporting that Ca^2+^ chelation as well as inhibition of phospholipase C, which acts downstream of P2RY2 (Figure 7), protect HT29 cells from dying due to extracellular ATP [50]. However, our results do not support findings suggesting that the activation of P2RY2 in CRC cells increases intracellular cAMP through Ca^2+^-dependent AC stimulation [50]. On the contrary, we found that HT29 cell exposure to extracellular ATP was associated with a modest but significant decrease in cAMP levels (Figure 6C) and that FSK-induced cAMP accumulation completely abolished cell death induction triggered by extracellular ATP (Figure 6D). This discrepancy might be explained by differential expression profiles of adenylyl cyclase coding genes and/or of the direct (Calmodulin, CaM) or indirect (CaM kinase, protein kinase C or calcineurin) modulators of ACs [84] existing in distinct HT29 subclones. In agreement with that hypothesis, it has been shown that extensive genetic heterogeneity exists within cultures of the same cancer cell lines, leading to global gene expression changes and striking differences in the response to anticancer drugs [85]. Our results therefore suggest that extracellular ATP stimulates phospholipase C and Ca^2+^ mobilization in HT29 cells through its binding to P2RY2, leading to a decrease in cAMP levels (Figure 7). This might in turn affect protein kinase A (PKA) and/or cAMP Response Element-binding protein (CREB) activities and modulate HT29 cell survival [86,87]. Further studies will be required in order to elucidate the precise roles of cAMP signaling downstream elements in HT29 cellular outcomes. Importantly, our model (Figure 7) is likely to account only for HT29 cells since no effect of the P2RY2 inhibitor AR-C 118925XX could be evidenced for the HCT116, LS513 and LS174T cell lines. Together, this makes it difficult to generalize a direct correlation between P2RY2 activation, Ca^2+^ mobilization, cAMP levels and cancer cell viability for CRC. Thus, targeting one specific pathway or receptor might prove difficult in terms of clinical practice.

Intriguingly, a common feature found in our four CRC cell lines is the ability of dipyridamole to impair cell death induction following extracellular ATP exposure (Figure 6E). DIP is a common anti-thrombotic agent [88] that exerts its pharmacological activities by inhibiting the reuptake of adenosine through its binding to equilibrative nucleoside transporters and/or by acting as a non-selective phosphodiesterase inhibitor, thus increasing cAMP and/or cGMP levels in cells [60,61,62]. Several reports showed that DIP blocks the effects of extracellular ATP on human gastric carcinoma HGC-27, histiocytic leukemia U-937 and cervical cancer cell survival by inhibiting adenosine uptake [83,89]. These results suggest that purinergic receptors may not play a major role in ATP and adenosine induced apoptosis in those cells. However, our data do not support this model for CRC, since DIP completely abolished the effects of both extracellular ATP and non-hydrolysable ATP on CRC cells (Figure 6E) while adenosine did not induce any form of cellular death at the concentrations tested here (Figure 4A and Figure 6E). Importantly, for HT29 as well as for LS174T cells, the effects of DIP confirm the outcome seen for FSK on cell survival (Figure 6D), suggesting that increasing cAMP levels might circumvent CRC cell response towards extracellular ATP (Figure 7). However, in LS174T cells the modulation of AC activity is likely not to rely on P2RY2 activation and Ca^2+^ mobilization (Figure 6A,B). Of note, AC activity can be directly and differentially modulated by protein kinase C (PKC) [84], a downstream target of P2Y1, P2Y2 and P2Y11 receptors which is expressed in all four CRC cell lines [15]. Moreover, it has been suggested that extracellular ATP might reduce Caco-2 CRC cells viability by inhibiting, not activating, PKC through an unknown ATP receptor [65]. Further investigation will be required to assess the role, if any, of PKC in our model (Figure 7). In addition to PKC modulation through Gα_q_ protein and phospholipase C activation, several P2Y receptors (P2Y12, P2Y13 and P2Y14) are known to bind Gα_i_ protein, which is capable of inhibiting adenylyl cyclases [15]. However, since none of those three receptors are expressed in our set of four CRC cell lines (Tables S1–S4), this hypothesis seems very unlikely in our setting (Figure 7). Finally, because DIP is a non-selective PDE inhibitor, cGMP levels might also be affected. This is important since both the cAMP and cGMP signaling pathways have been found to have either positive or negative effects on cancer cell growth and survival [90]. Moreover, several works have reported that in different tissues extracellular ATP can induce nitric oxide signaling, the target enzyme of which is soluble guanylyl cyclase [91,92]. The involvement of cGMP modulation may account for the HCT116 and LS513 cells for which FSK effect could not be evaluated. However, even though this quite unexplored area may seem far beyond the scope of our current study, it does suggests that differential signaling pathways as well as different purinergic receptors might be activated in CRC, leading to distinctive cellular outcomes [43].

## 4. Materials and Methods

### 4.1. Cells and Therapeutics

Human colorectal carcinoma cells (HT29, LS513, LS174T and HCT116) were kindly provided by Richard Camalier (Division of Cancer Treatment and Diagnosis Tumor Repository, National Cancer Institute) and Richard Hamelin (Paris, France). Colon carcinoma cell lines were maintained in DMEM (except for HCT116 cell lines, which were cultured in McCoy medium) supplemented with 5% FBS and 100 units/mL penicillin and 100 µg/mL streptomycin. The FHC cell line (established from normal human fetal colonic mucosa) was obtained from ATCC (American Type Culture Collections, VA, USA), while the hTERT and cdk4 immortalized human colon epithelial cell line (HCEC-1CT) was purchased from Evercyte. Both FHC and HCEC-1CT cell lines were maintained as described by the suppliers.

ATP (Adenosine 5′-trisphosphate disodium salt hydrate), ATPγS (Adenosine 5′-[γ-thio]triphosphate tetralithium salt), adenosine, QVD-Oph, Necrostatin-1 and dipyridamole (DIP) were purchased from Merck. MRS 2179 and AR-C 118925XX were purchased from Tocris, while forskolin (FSK) was obtained from abcam.

### 4.2. RNA Extraction and RNA Sequencing

Whole RNA was extracted from the four CRC cell lines grown in either 2D or 3D conditions by using the RNeasy^®^ Plus Micro Kit (Qiagen), as described by the supplier. Library preparation and Illumina sequencing were performed at the Ecole Normale Superieure genomics core facility (Paris, France). Briefly, messenger (polyA+) RNAs were purified from 300 ng of total RNA using oligo(dT). Libraries were prepared using the strand specific RNA-Seq library preparation TruSeq Stranded mRNA kit (Illumina). Libraries were multiplexed by 24 on two flowcells. A 75 bp single read sequencing was performed on a NextSeq 500 (Illumina). A mean of 36.7 ± 1.3 million passing Illumina quality filter reads was obtained for each of the 24 samples. The analyses were performed using the Eoulsan pipeline [93], including read filtering, mapping, alignment filtering, read quantification, normalization and differential analysis; before mapping, adapters were removed using Trim Galore! (Version 0.4.1) (https://www.bioinformatics.babraham.ac.uk/projects/trim_galore/, accessed on 12 October 2021), poly N read tails were trimmed, reads with ≤40 bases were removed, and reads with a quality of mean ≤30 were discarded. Reads were then aligned against the Homo sapiens genome from Ensembl version 91 using STAR (version 2.6.1b) [94]. Alignments from reads matching more than once on the reference genome were removed using the Java version of samtools [95]. To compute gene expression, Homo sapiens GTF genome annotation version 91 from the Ensembl database was used. All overlapping regions between alignments and referenced exons were counted and aggregated by genes using HTSeq-count 0.5.3 [96]. The sample counts were normalized using DESeq2 1.8.1 [97]. Statistical treatments and differential analyses were also performed using DESeq2 1.8.1. Finally, the hierarchical cluster plot was performed on normalized counts with the hcust function of the “stats” package of R 3.2.2 using the “complete” agglomeration method. The RNASeq gene expression data and raw fastq files are available on the GEO repository (www.ncbi.nlm.nih.gov/geo/, accessed on 12 October 2021) under accession number: GSE185055.

### 4.3. Quantitative Real Time PCR

Total RNA was extracted as described above using the RNeasy^®^ Plus Micro Kit (Qiagen). cDNA was prepared using RevertAid H Minus Reverse Transcriptase (ThermoFisher). Quantitative real-time PCR (qRT-PCR) was performed in duplicate using SYBR Green Gene Expression Assays (SensiFAST^TM^ SYBR^®^ No-Rox Kit Bioline). Reactions were analyzed with a Biorad CFX96 Touch Real-Time PCR machine at 62 °C for 40 cycles. Data were analyzed using the comparative threshold cycle 2^−ΔΔCt^ method [98]; the expression of 36B4 (RPL0) was used to normalize data. The expression of the genes was expressed as the fold change in the samples compared to HCEC-1CT cells; 2^−ΔΔCt^ calculation for three independent genes is available Table S5. All values are averages of at least four independent experiments done in duplicate. Gene specific primers, KiCqStart^®^ SYBR^®^ Green Primers, were purchased from Sigma Aldrich-Merck and are listed as follows. 


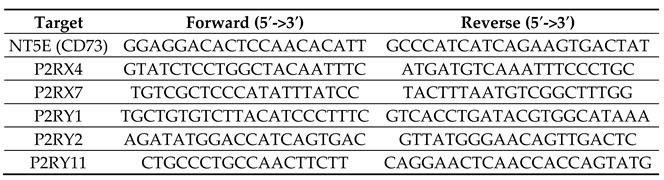


### 4.4. Cell Viability Assay

Cell viability was assessed by CellTiter Glo Luminescent Viability assay (Promega). At J0, 1500 cells were seeded in a white 96-well plate. At J1, cells were treated with ATP, ATPγS or adenosine at the indicated concentrations. After four days of treatment, a CellTiter Glo assay was performed as recommended by Promega. Luminescence was measured in a microplate reader, Infinite 200Pro (TECAN). All values are averages of at least three independent experiments done in duplicate. The IC50 values were calculated with the Graphpad Prism software using a nonlinear regression curve.

### 4.5. Spheroid Growth

Cells were seeded in a low attachment plate (Corning), specific for spheroid formation; 2000 cells were plated, and growth was assessed after eight days by microscopy (Evos, inverted microscope). Cells were treated after 24 h of culture with ATP (250 µM) and adenosine (250 µM). Image analysis was performed using ImageJ software to determine the area growth of the spheroids.

### 4.6. Cell Death Induction and Inhibition

At J0, 5 × 10^4^ cells were seeded in a 24-well plate with 500 µL of medium. At J1, medium was replaced by fresh pre-warmed medium complemented (or not) with the inhibitors QVD-Oph (pan-caspases inhibitor, 10 µM), Necrostatin-1 (RIPK1 inhibitor, 40 µM), MRS 2179 (competitive P2RY1 antagonist, 10 µM), AR-C 118925XX (competitive P2RY2 antagonist, 4 µM), FSK (adenylyl cyclase activator, 100 µM), DIP (non-selective phosphodiesterase inhibitor, 10 µM). After 30 min, cells were treated with ATP (250 µM), ATPγS (250 µM) or adenosine (250 µM) and left for 48 h. At J3, medium was collected in a tube. Cells were then washed with PBS, harvested with Accutase (Merck) and collected in the same tube. Cells were double stained with Annexin-V-APC (0.1 µg/mL; BD Biosciences) and propidium iodide (PI, 0.5 µg/mL) to assess phosphatidyl-serine (PS) exposure and cell viability, respectively. Cell death induction was recorded for the total population (10^4^ cells) in a cytoFLEX (Beckman Coulter) and data were analyzed using FlowJo software.

### 4.7. γ-H2AX Immunolabeling

At J0, 5 × 10^4^ cells were seeded in a 24-well plate with 500µL of medium. At J1, cells were treated with ATP (250µM) or adenosine (250µM) for 48 h. The DNA double stranded breaks (DSBs) formation was then assessed by the measurement of Ser-139 phosphorylation of the histone variant H2AX with an indirect labeling protocol. Cells were fixed and permeabilized by PFA (4%) and then ice-cold methanol, and labeled by the primary antibody anti-γ-H2AX (ab26350—Abcam, 1µg/10^6^ cells diluted in PBS-BSA 1%). Then, cells were incubated with an anti-mouse FITC-conjugated secondary antibody (concentration 1/250), and the signal was measured by flow cytometry using a cytoFLEX (Beckman coulter). Data were analyzed using FlowJo software and the percentage of γ-H2AX positive cells determined.

### 4.8. Cell Cycle Analysis

Cell cycle analysis was performed by using the APC BrdU Flow kit (BD biosciences). Briefly, 10^5^ cells were treated by ATP (250 µM) for 24 h and incubated with 10 µM bromodeoxyuridine (BrdU) for 1 h at 37 °C. Cells were then fixed and stained with an APC-conjugated anti-BrdU antibody and 7-aminoactinomycin D (7-AAD) according to the manufacturer’s instructions. Two-color flow cytometry was performed in a cytoFLEX (Beckman Coulter) and data analyzed to identify the percentage of cells actively synthesizing DNA (BrdU incorporation) and their cell cycle position (i.e., G0/1, S, or G2/M phase defined by 7-AAD staining intensities).

### 4.9. Measurement of Intracellular Calcium Concentrations

At J0, 8.10^4^ cells were seeded into a black 96-well plate with 200 µL of medium. At J1, cells were washed with HEPES-buffered saline (HBS) solution (145 mM NaCl, 5 mM KCl, 1 mM CaCl_2_, 1 mM MgCl_2_, 10 mM Glucose, 10 mM HEPES, pH 7.4) and loaded for 1 h at room temperature with 5 µM Fura2-AM and 0.05% Pluronic acid (ThermoFisher). The cells were then washed twice in HBS buffer and treated (or not) with MRS 2179 or AR-C 118925XX. Fluorescence emission was recorded at 510 nm on a microplate reader, Infinite 200pro (TECAN), after cell exposure to 340 and 380 nm wavelengths of exciting light. After 10 min of basal measurement, cells were exposed to either ATP (250 µM) or ionomycin (1 µM, positive control) and fluorescence recorded for 30 additional minutes. Signals were computed into the 340/380 nm excitation ratio to allow accurate measurements of the intracellular Ca^2+^ concentration. Data were normalized, with the mean values calculated for the first 10 min. All values are averages of four independent experiments done in duplicate.

### 4.10. cAMP Levels Measurement

Cyclic adenosine monophosphate (cAMP) cellular levels were assessed by cAMP Direct Immunoassay Detection Kit (Abcam). Briefly, at J0, 5.10^4^ cells were seeded into a 96-well plate. At J1, cells were treated (or not) with ATP (250 µM) for either 5 or 15 min. cAMP measurement was then performed according to the manufacturer’s instructions. Florescence (Ex/Em: 540/590 nm) was measured in a microplate reader Infinite 200Pro (TECAN). All values are averages of four independent experiments.

### 4.11. Statistical Analysis

Student’s *t*-test was performed using GraphPad Prism (GraphPad Software). Statistically significant *p*-values were indicated as follows: *: *p* ≤ 0.05, **: *p* ≤ 0.01, ***: *p* ≤ 0.001.

## Data Availability

The RNASeq gene expression data are available at www.ncbi.nlm.nih.gov/geo/ (accessed on 12 October 2021) under the accession number: GSE185055.

## Supplementary Materials

The following are available online at https://www.mdpi.com/article/10.3390/ijms222111472/s1.
