# Supplementary material for: Expression Pattern of Purinergic Signaling Components in Colorectal Cancer Cells and Differential Cellular Outcomes Induced by Extracellular ATP and Adenosine
Source: Int J Mol Sci. 2021 Oct 25;22(21):11472. doi: 10.3390/ijms222111472 (PMC8583864; doi:10.3390/ijms222111472)

Supplemental information

Quantitative Real Time PCR

As described in the main text of the manuscript, total RNA was extracted using the RNeasy® Plus Micro Kit (Qiagen), cDNA was prepared using RevertAid H Minus Reverse Transcriptase (ThermoFisher) and qRT-PCR was performed in duplicate using SYBR Green Gene Expression Assays (SensiFAST™ SYBR® No-Rox Kit Bioline). Data were analysed using the comparative threshold cycle method with 36B4 gene expression levels used for data normalization. Gene specific primers, KiCqStart® SYBR® Green Primers, were purchased from Sigma Aldrich-Merck and are listed thereafter:

Target	Forward (5’->3’)	Reverse (5’->3’)
CD39	CTTGTGCTATGGGAAGGATCAG	GCATGGGTCCCTGAGAATTT
P2RX1	CCTAAGAGGCACTACTACAAG	ATCAGGATGTCCTCATGTTC
P2RX2	CCTCTGTCAGCCAATTTC	ATTTGGGGTAGTGGATGC
P2RX6	CAGAACTTCACACTGTTCATC	GGCTGAATTGTGGTTCATAG
P2RY4	AGGGAACCCAATAGTGATAC	GAGTAGAAGATTGGCATTGG
P2RY12	AAGAGCACTCAAGACTTTAC	GGGTTTGAATGTATCCAGTAAG
P2RY13	ACAGAGAGAACTGAGTATCC	CACAGAGCCAAAGTATTCAG

Supplementary table S1

Expression levels of selected genes involved in purinergic signaling: *HT29 cells were grown in either 2D or 3D culture conditions and gene expression levels determined through RNA sequencing as described in the main core of the manuscript. Boxed lines indicate the genes consistently expressed in all the CRC cell lines tested.*

		Counts 2D	Counts 3D	Log2foldchange 2Dvs3D	pvalue
ENTPD1 (CD39)	ENSG00000138185	0,583	0,708	0,17241803	0,865473277
NT5E (CD73)	ENSG00000135318	268,250	162,500	-0,91419885	2,01149E-07
PANX1	ENSG00000110218	101,833	103,000	-0,21249751	0,247035108
P2RX1	ENSG00000108405	0,042	0,542	2,82761758	0,006551099
P2RX2	ENSG00000187848	0,000	0,000		
P2RX3	ENSG00000109991	0,000	0,000		
P2RX4	ENSG00000135124	21,458	64,375	1,33932901	6,98108E-14
P2RX5	ENSG00000083454	0,250	0,042	-1,31357412	0,065296492
P2RX6	ENSG00000099957	2,500	6,417	1,15510213	0,00772777
P2RX7	ENSG00000089041	0,083	0,042	-0,4427451	0,659749943
P2RY1	ENSG00000169860	6,667	5,375	-0,58119221	0,077381602
P2RY2	ENSG00000175591	119,417	133,042	-0,0722265	0,704047614
P2RY4	ENSG00000186912	0,000	0,000		
P2RY6	ENSG00000171631	0,083	0,125	0,12065394	0,934306669
P2RY11	ENSG00000244165	36,292	48,625	0,19078382	0,446042779
P2RY12	ENSG00000169313	0,000	0,000		
P2RY13	ENSG00000181631	0,000	0,000		
P2RY14	ENSG00000174944	0,000	0,000		
A1	ENSG00000163485	0,083	0,250	0,62790844	0,452658535
A2A	ENSG00000128271	1,292	1,667	0,1479647	0,789833651
A2B	ENSG00000170425	29,875	28,042	-0,31886031	0,022922839
A3	ENSG00000282608	0,000	0,083		

Total counts 2D	3329252,79
Total counts 3D	3968994,54

Supplementary table S2

***Expression levels of selected genes involved in purinergic signaling:** LS513 cells were grown in either 2D or 3D culture conditions and gene expression levels determined through RNA sequencing as described in the main core of the manuscript. Boxed lines indicate the genes consistently expressed in all the CRC cell lines tested.*

		Counts 2D	Counts 3D	Log2foldchange 2Dvs3D	pvalue
ENTPD1 (CD39)	ENSG00000138185	0,042	0,042	0,27594853	0,679040738
NT5E (CD73)	ENSG00000135318	176,042	118,708	-0,39115783	0,015786378
PANX1	ENSG00000110218	109,833	90,083	-0,11401145	0,626998453
P2RX1	ENSG00000108405	0,208	1,167	2,61257177	0,001459872
P2RX2	ENSG00000187848	0,000	0,000		
P2RX3	ENSG00000109991	0,000	0,042	1,47749557	0,614491146
P2RX4	ENSG00000135124	56,750	106,500	1,08213624	9,24759E-12
P2RX5	ENSG00000083454	0,000	0,000		
P2RX6	ENSG00000099957	4,042	4,667	0,48401614	0,187739029
P2RX7	ENSG00000089041	0,042	0,000		
P2RY1	ENSG00000169860	76,458	56,375	-0,28829386	0,210654481
P2RY2	ENSG00000175591	59,458	90,500	0,76920493	1,03808E-08
P2RY4	ENSG00000186912	0,042	0,083	0,2547531	0,819025089
P2RY6	ENSG00000171631	0,042	0,000	-0,22993798	0,791807641
P2RY11	ENSG00000244165	37,958	39,333	0,22918604	0,221955153
P2RY12	ENSG00000169313	0,000	0,000		
P2RY13	ENSG00000181631	0,000	0,000		
P2RY14	ENSG00000174944	0,000	0,000		
A1	ENSG00000163485	0,083	0,000	-0,40337452	0,480024752
A2A	ENSG00000128271	0,333	0,625	0,79351517	0,109065557
A2B	ENSG00000170425	247,958	229,458	0,06367985	0,505226793
A3	ENSG00000282608	0,000	0,000		

Total counts 2D	3994063,12
Total counts 3D	3911649,67

Supplementary table S3

Expression levels of selected genes involved in purinergic signaling: *HCT116 cells were grown in either 2D or 3D culture conditions and gene expression levels determined through RNA sequencing as described in the main core of the manuscript. Boxed lines indicate the genes consistently expressed in all the CRC cell lines tested.*

		Counts 2D	Counts 3D	Log2foldchange 2Dvs3D	pvalue
ENTPD1 (CD39)	ENSG00000138185	0,833	0,625	-0,01075877	0,993151258
NT5E (CD73)	ENSG00000135318	131,750	211,375	0,78465174	1,72066E-05
PANX1	ENSG00000110218	139,000	110,167	-0,19321148	0,32554193
P2RX1	ENSG00000108405	0,000	0,000		
P2RX2	ENSG00000187848	0,000	0,000		
P2RX3	ENSG00000109991	0,000	0,083		
P2RX4	ENSG00000135124	41,458	70,500	0,89850821	5,98902E-07
P2RX5	ENSG00000083454	69,458	59,750	-0,08944247	0,606801488
P2RX6	ENSG00000099957	0,083	0,625	1,97177013	0,003244549
P2RX7	ENSG00000089041	7,792	7,042	-0,03147187	0,962904477
P2RY1	ENSG00000169860	10,792	5,167	-0,89983104	0,00419208
P2RY2	ENSG00000175591	21,667	17,333	-0,17668704	0,422941595
P2RY4	ENSG00000186912	0,125	0,208	0,26723185	
P2RY6	ENSG00000171631	0,958	0,750	-0,09958365	0,948214301
P2RY11	ENSG00000244165	97,333	106,625	0,25908546	0,292485259
P2RY12	ENSG00000169313	0,000	0,000		
P2RY13	ENSG00000181631	0,000	0,000		
P2RY14	ENSG00000174944	0,042	0,000		
A1	ENSG00000163485	26,917	21,917	-0,15445415	0,656069292
A2A	ENSG00000128271	25,667	19,250	-0,25731977	0,430194994
A2B	ENSG00000170425	176,917	193,667	0,26508206	0,02426093
A3	ENSG00000282608	0,000	0,000		

Total counts 2D	3868643,00
Total counts 3D	3805645,62

Supplementary table S4

Expression levels of selected genes involved in purinergic signaling: *LS174T cells were grown in either 2D or 3D culture conditions and gene expression levels determined through RNA sequencing as described in the main core of the manuscript. Boxed lines indicate the genes consistently expressed in all the CRC cell lines tested.*

		Counts 2D	Counts 3D	Log2foldchange 2Dvs3D	pvalue
ENTPD1 (CD39)	ENSG00000138185	0,292	0,625	0,73792444	0,42963845
NT5E (CD73)	ENSG00000135318	200,250	153,708	-0,23863167	0,27297981
PANX1	ENSG00000110218	97,375	77,875	-0,19119169	0,33789564
P2RX1	ENSG00000108405	0,000	0,000		
P2RX2	ENSG00000187848	0,000	0,000		
P2RX3	ENSG00000109991	0,042	0,000		
P2RX4	ENSG00000135124	26,667	35,167	0,5777736	0,00469974
P2RX5	ENSG00000083454	54,708	43,292	-0,18892671	0,23265389
P2RX6	ENSG00000099957	1,083	1,208	0,48511635	0,43616165
P2RX7	ENSG00000089041	0,042	0,083	0,11610692	0,92498638
P2RY1	ENSG00000169860	41,333	24,583	-0,62297166	0,0344497
P2RY2	ENSG00000175591	16,875	15,167	-0,00901346	0,97649341
P2RY4	ENSG00000186912	0,167	0,042		
P2RY6	ENSG00000171631	0,000	0,042		
P2RY11	ENSG00000244165	87,375	89,583	0,18700387	0,46940936
P2RY12	ENSG00000169313	0,000	0,000		
P2RY13	ENSG00000181631	0,000	0,000		
P2RY14	ENSG00000174944	0,000	0,000		
A1	ENSG00000163485	0,292	0,375	0,36013038	0,67439652
A2A	ENSG00000128271	0,750	0,667	0,0261776	0,97511343
A2B	ENSG00000170425	194,708	184,625	0,06708552	0,64613996
A3	ENSG00000282608	0,000	0,000		

Total counts 2D	3867423,12
Total counts 3D	3807228,46

Supplementary table S5

Transcriptional expression levels of 3 independent genes (A, NT5E; B, PANX1; C, P2Y2) measured by quantitative Real-Time PCR in CRC (HT29, LS513, HCT116 and LS174T) and non-tumorigenic colonic (HCEC-1CT) cell lines (n =4) grown in 2D. *Fold changes were calculated by using the $2^{-\Delta\Delta Ct}$ method. Briefly, for each cell line, the mRNA expression levels of the housekeeping gene 36B4 (RPL0) were used as an internal control to normalize gene expression levels. The difference between the Ct values (ΔCt) of the genes of interest and the housekeeping gene was then calculated for each experimental sample and the difference in the ΔCt values between the experimental (CRC cells) and control (HCEC-1CT) samples ΔΔCt calculated. Relative gene expression levels were expressed as the fold changes calculated for each CRC samples compared to the HCEC-1CT cells (equal to $2^{-\Delta\Delta C}$).*

All Ct values are averages of at least 4 independent experiments done in duplicate.

(A)

Cell lines	Genes	Ct	ΔCt = Ct gene of interest - Ct housekeeping gene	ΔΔCt = ΔCt cells of interest - ΔCt control HCEC1-CT cells	Fold changes = $2^{-\Delta\Delta Ct}$
HCT116	**NT5E**	20,04	6,56	2,095	0,234068062
HT29		19,62	6,07	1,605	0,32873569
LS513		18,92	5,25	0,785	0,580351957
LS174T		19,491	6,361	1,896	0,268687293
HCEC-1CT		17,515	4,465	0	1
HCT116	**RPL0**	13,48			
HT29		13,55			
LS513		13,67			
LS174T		13,13			
HCEC-1CT		13,05			

(B)

Cell lines	Genes	Ct	ΔCt = Ct gene of interest - Ct housekeeping gene	ΔΔCt = ΔCt cells of interest - ΔCt control HCEC1-CT cells	Fold changes = $2^{-\Delta\Delta Ct}$
HCT116	**PANX1**	19,14	6,12	0,712	0,610473256
HT29		19,35	5,98	0,572	0,672683604
LS513		19,25	5,4975	0,0895	0,939848419
LS174T		19,4525	6,3725	0,9645	0,512455984
HCEC-1CT		17,748	5,408	0	1
HCT116	**RPLO**	13,02			
HT29		13,37			
LS513		13,7525			
LS174T		13,08			
HCEC-1CT		12,34			

(C)

Cell lines	Genes	Ct	ΔCt = Ct gene of interest - Ct housekeeping gene	ΔΔCt = ΔCt cells of interest - ΔCt control HCEC1-CT cells	Fold changes = $2^{-\Delta\Delta Ct}$
HCT116	**P2Y2**	24,425	10,705	-11,775	3504,517225
HT29		20,9	7,46	-15,02	33225,42423
LS513		21,88	8,1	-14,38	21321,18496
LS174T		23,64	10,31	-12,17	4608,239553
HCEC-1CT		35,17	22,48	0	1
HCT116	**RPLO**	13,72			
HT29		13,44			
LS513		13,78			
LS174T		13,33			
HCEC-1CT		12,69			

Supplementary Figure S1

Correlation of gene expression levels assessed on extracts prepared from cells grown in 2D through RNA sequencing (Counts, Y axis) and qRTPCR (Ct values, X axis).

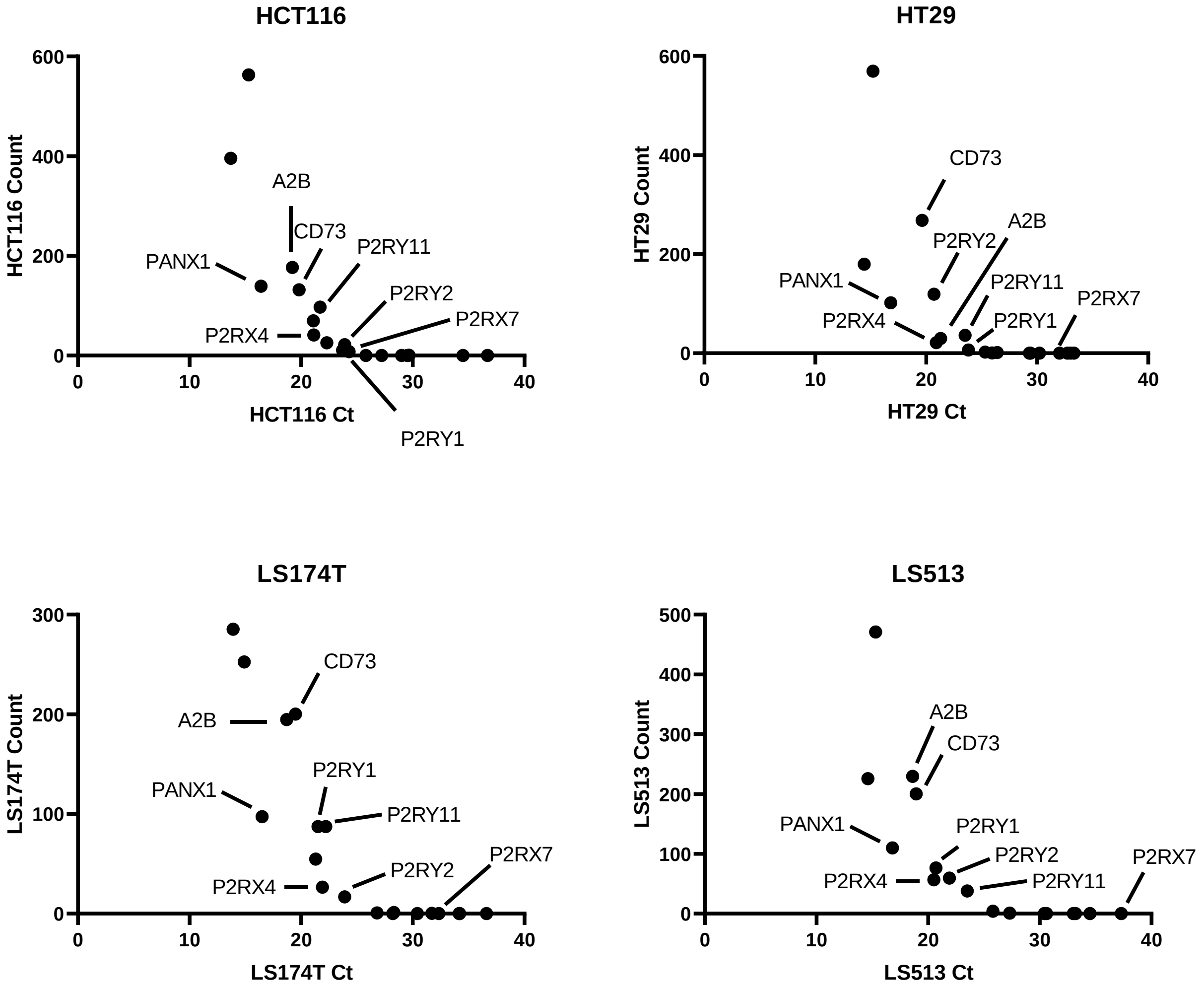

Supplementary Figure S2

Effect of purine molecules on 2D cell viability. *CRC (HCT116, LS513, HT29, LS174T) and non-tumorigenic colonic (FHC, HCEC-1CT) cell lines were treated for 4 days with increasing concentrations of ATP, ATPγS and adenosine (range 0-1000 µM) and cell viability assessed by the MTT assay (upper panels; n ≥ 3). IC50 mean values were calculated and are indicated in the lower panels (histograms and table).*

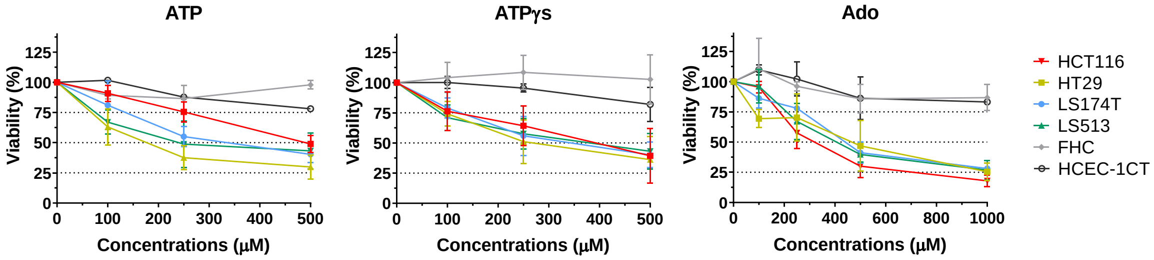

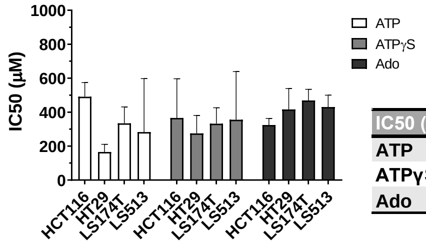

IC50 (µM)	HCT116	HT29	LS174T	LS513	FHC	HCEC-1CT
**ATP**	490.7	165.3	333.9	282.5	>1000	>1000
**ATPγS**	365.7	274.7	331.8	355.4	>1000	>1000
**Ado**	324	416.2	469.2	430.4	>1000	>1000

Supplementary Figure S3

Effect of purine molecules on Cell cycle progression. *(A) Flow cytometry cell cycle analysis carried out on CRC cell lines treated by ATP (250µM – 24h) or Adenosine (250µM – 24h) by BrdU and 7-AAD (DNA content) co-labeling. On this graph, only the percentage of cells in S phase is represented. (B) Flow cytometry representative diagrams obtained after treatment of LS174T cells with ATP (250µM), Adenosine (250µM) or left untreated for 24h and then labeled with BrdU and 7-AAD (DNA content). (C) Flow cytometry representative diagrams obtained after treatment of HT29 cells with ATP (250µM), Adenosine (250µM) or left untreated for 24h and then labeled with BrdU and 7-AAD (DNA content).*

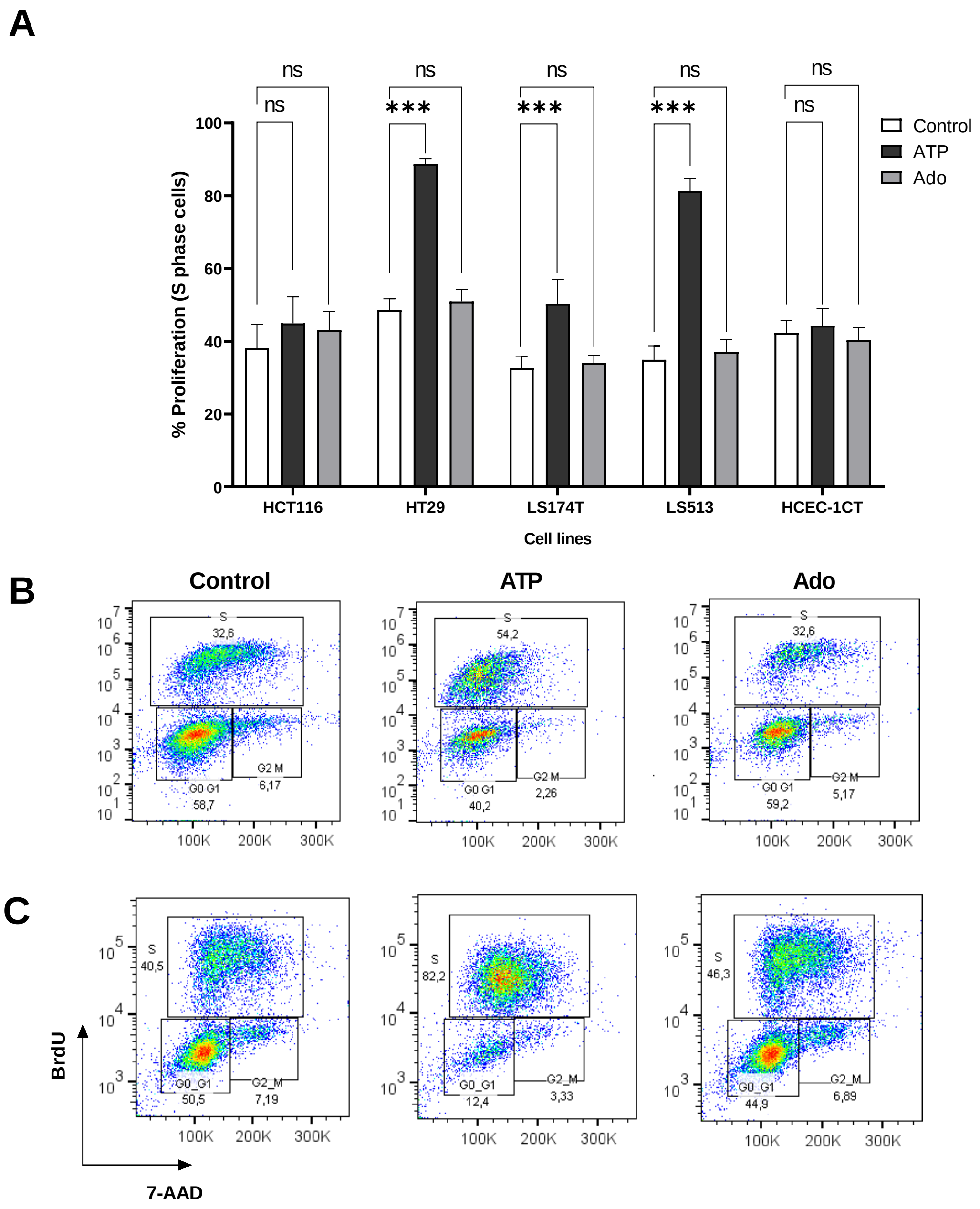

Supplementary Figure S4

Effect of the AR-C 118925XX competitive P2RY2 receptor antagonist on ATP anticancer activity. *HT29 and HCEC-1CT cell lines were treated for 4 days with increasing concentrations of ATP (range 0-1000 µM) in presence or not of 4 µM AR-C 118925XX. Cell viability was assessed by the CellTiter-Glo 2.0 assay. Data are expressed as mean +/- SD (n = 3). SDs are indicated by error bars when they exceed symbol size.*

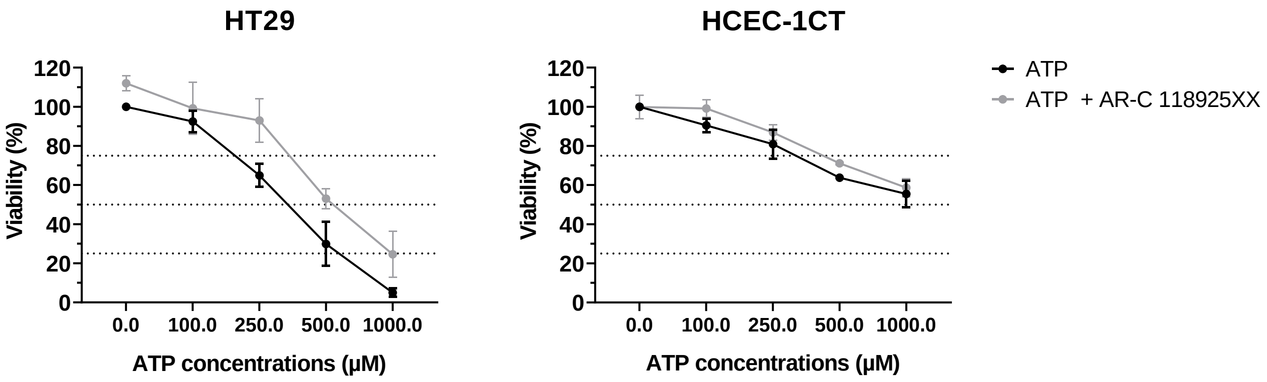

Supplementary Figure S5

Effect of Forskolin on cAMP levels in CRC cells. *CRC (HCT116, HT29, LS174T, LS513) and normal-like colonic (HCEC-1T) cells were treated or not for 15 minutes with the Adenylyl cyclase activator (FSK, 100 µM) and cAMP levels determined by the cAMP Direct Immunoassay Detection Kit (Abcam).*

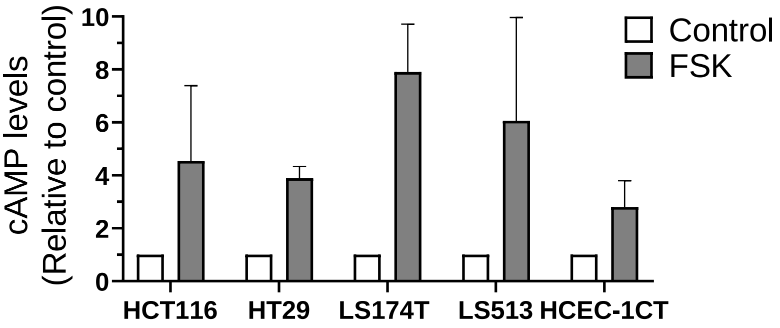

Supplementary Figure S6

Effect of Forskolin on ATP anticancer activity. *CRC (HCT116, LS513) cells were treated or not for 30 minutes with the Adenylyl cyclase activator (FSK, 100 µM) and then incubated with 250 µM ATP for 48 hours. The percentage of dead cells (Annexin V+) was determined by flow cytometry. Data are expressed as mean +/- SD (n = 4).*

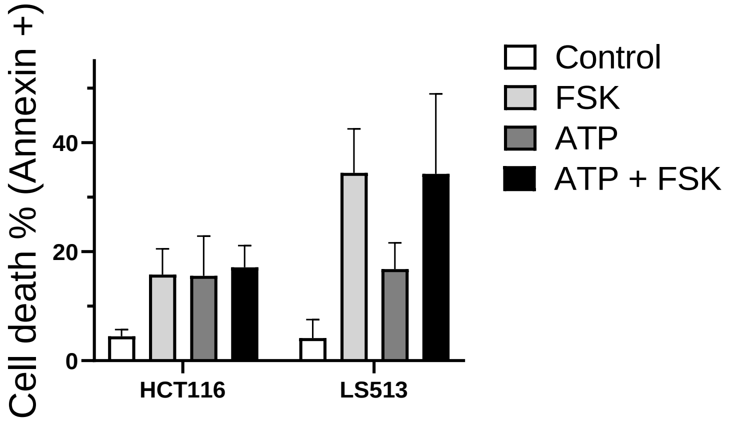

Supplement: Supplementary file 1 [file ijms-22-11472-s001.zip › ijms-1432758-supplementary.pdf]
